# Distribution of proteins within different compartments of tendon varies according to tendon type

**DOI:** 10.1111/joa.12485

**Published:** 2016-04-25

**Authors:** Chavaunne T. Thorpe, Kabelan J. Karunaseelan, Jade Ng Chieng Hin, Graham P. Riley, Helen L. Birch, Peter D. Clegg, Hazel R. C. Screen

**Affiliations:** ^1^ Institute of BioengineeringSchool of Engineering and Materials ScienceQueen Mary University of LondonLondonUK; ^2^ School of Biological SciencesUniversity of East AngliaNorwich Research ParkNorwichUK; ^3^ Institute of Orthopaedics and Musculoskeletal ScienceUniversity College LondonRoyal National Orthopaedic HospitalStanmoreUK; ^4^ Department of Musculoskeletal BiologyInstitute of Ageing and Chronic DiseaseUniversity of LiverpoolNestonUK

**Keywords:** endotenon, histology, immunohistochemistry, interfascicular matrix, proteoglycans

## Abstract

Although the predominant function of all tendons is to transfer force from muscle to bone and position the limbs, some tendons additionally function as energy stores, reducing the energetic cost of locomotion. To maximise energy storage and return, energy‐storing tendons need to be more extensible and elastic than tendons with a purely positional function. These properties are conferred in part by a specialisation of a specific compartment of the tendon, the interfascicular matrix, which enables sliding and recoil between adjacent fascicles. However, the composition of the interfascicular matrix is poorly characterised and we therefore tested the hypothesis that the distribution of elastin and proteoglycans differs between energy‐storing and positional tendons, and that protein distribution varies between the fascicular matrix and the interfascicular matrix, with localisation of elastin and lubricin to the interfascicular matrix. Protein distribution in the energy‐storing equine superficial digital flexor tendon and positional common digital extensor tendon was assessed using histology and immunohistochemistry. The results support the hypothesis, demonstrating enrichment of lubricin in the interfascicular matrix in both tendon types, where it is likely to facilitate interfascicular sliding. Elastin was also localised to the interfascicular matrix, specifically in the energy‐storing superficial digital flexor tendon, which may account for the greater elasticity of the interfascicular matrix in this tendon. A differential distribution of proteoglycans was identified between tendon types and regions, which may indicate a distinct role for each of these proteins in tendon. These data provide important advances into fully characterising structure–function relationships within tendon.

## Introduction

Energy‐storing tendons such as the human Achilles and patellar tendons have an important role in reducing the energetic cost of locomotion by stretching and recoiling with each stride to store and return energy (Lichtwark & Wilson, 2005; Malliaras et al. 2015). To enable this function, they have distinct mechanical properties such as greater extensibility and elasticity leading to improved energy storage, when compared with tendons that are purely positional in function, such as the anterior tibialis tendon (Maganaris & Paul, 1999; Batson et al. 2003; Lichtwark & Wilson, 2005; Thorpe et al. 2012).

**Figure 1:**
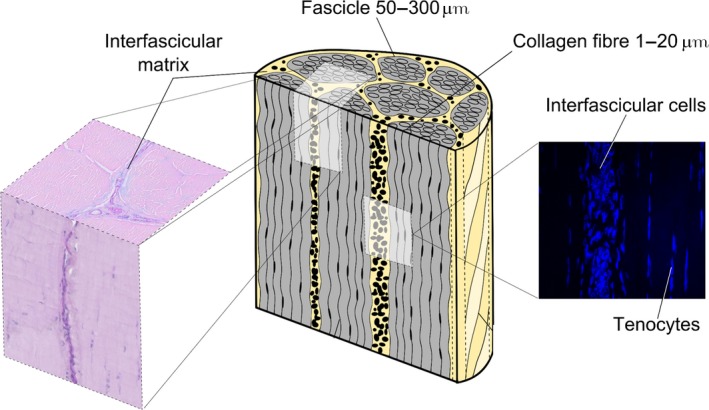
Schematic showing the hierarchical structure of tendon in which collagen aggregates to form subunits of increasing diameter, the largest of which is the fascicle. Fascicles are interspersed by the interfascicular matrix (IFM, also known as the endotenon). Reproduced from Thorpe et al., 2015a,b with kind permission from Wiley publications.

The specific mechanical properties required for optimum energy storage are conferred by specialised structure and composition of energy‐storing tendons. All tendons have the same basic structure, in which highly aligned collagen molecules are grouped together in a hierarchical manner, forming subunits of increasing diameter, the largest of which is the fascicle (Fig. 1) (Kastelic et al. 1978). At the higher hierarchical levels, the collagenous subunits are interspersed with a less fibrous, hydrated matrix, traditionally referred to as ground substance (for more details see Thorpe et al. 2013a and references therein). Small alterations in structure and composition throughout the hierarchy are thought to contribute to the gross differences in mechanical properties; for example, it has been shown that the collagen crosslink profile and collagen fibril diameter differ in energy‐storing and positional tendons (Birch et al. 2006; Birch, 2007). It has also been demonstrated that fascicle structure differs between tendon types, with the presence of a helical component in fascicles from energy‐storing tendons which enables increased elasticity (Thorpe et al. 2013b). Further, our previous studies have demonstrated that the matrix interspersing fascicles [the interfascicular matrix (IFM)] is more extensible and elastic in energy‐storing tendons than in positional tendons, providing the capacity for sliding and recoil between fascicles and therefore enabling the greater extensions required by energy‐storing tendons (Thorpe et al. 2012, 2015b). The composition of the IFM, however, is poorly characterised, and very little is known about compositional specialisation and how this results in the distinct mechanical properties seen in the IFM of energy‐storing tendons.

One of the few previous studies to investigate the composition of the IFM characterised the proteome of the IFM and fascicular matrix (FM) in an energy‐storing tendon. This study demonstrated that the IFM has a distinct proteome, with a greater number of proteins identified in the IFM than in the FM (Thorpe et al. 2016). Other studies have also shown that the IFM is rich in lubricin (also known as superficial zone, or proteoglycan‐4) (Funakoshi et al. 2008; Sun et al. 2015) and elastin (Smith et al. 2011; Grant et al. 2013). Lubricin is a heavily glycosylated protein that has both glycoprotein and proteoglycan isoforms (Lord et al. 2011). In joints, lubricin provides boundary layer lubrication (Lord et al. 2011). In tendon, it has been shown previously that interfascicle gliding is impaired in the tail tendons of lubricin null mice (Kohrs et al. 2011), and fascicle viscoelastic properties are altered with lubricin depletion (Reuvers et al. 2011). Elastin is a highly extensible, fatigue‐resistant fibrous protein that is present in tissues that are subjected to high levels of cyclic loading, including arteries and heart valves (Gosline et al. 2002; Lillie & Gosline, 2002). However, no previous studies have directly compared the distribution of lubricin and elastin in energy‐storing and positional tendons.

While it is well established that the small leucine‐rich proteoglycans (SLRPs) decorin, biglycan, fibromodulin and lumican are present in tendon and that they play an important role in regulation of collagen fibrillogenesis during tendon development (Thorpe et al. 2013a), differences in their distribution in functionally distinct tendons, specifically their distribution in the FM and IFM, have not been studied.

The horse is a relevant and accepted model for tendon research, as it is an athletic species which maximises energy efficiency by storage and release of elastic energy in the limb tendons. The predominant energy store in the horse is the forelimb superficial digital flexor tendon (SDFT), which has an analogous function to the Achilles tendon (Biewener, 1998; Innes & Clegg, 2010; Lui et al. 2010). Indeed, tendon injuries in the SDFT show a very similar epidemiology, aetiology and pathology to those seen in human Achilles tendon injuries (Innes & Clegg, 2010; Lui et al. 2010). The anatomically opposing equine common digital flexor tendon (CDET) is an example of a positional tendon, functionally comparable to the human anterior tibialis tendon (Birch, 2007).

In this study, we assessed the distribution of a number of matrix proteins within the equine SDFT and CDET using histology and immunohistochemistry. We hypothesised that the distribution of elastin and proteoglycans would differ between the tendon types. We further hypothesised that protein distribution would vary between the fascicular and interfascicular matrices, with localisation of elastin and lubricin to the IFM.

## Materials and methods

### Sample collection

Forelimbs, distal to the carpus, were collected from horses aged 3–7 years euthanised for reasons unrelated to this project (*n* = 5). The SDFT and CDET were harvested from each forelimb within 24 h of euthanasia. Any tendons with macroscopic evidence of injury were excluded from the study. Sections (approximately 15 × 5 × 5 mm) were removed from the mid‐metacarpal region of each tendon and fixed for 24 h in 4% paraformaldehyde in phosphate‐buffered saline at 4 °C. Samples were then paraffin‐embedded, and 4‐μm‐thick serial transverse and longitudinal sections were cut from each sample and attached to positively charged slides.

### Histology

To assess the general distribution of proteoglycans and glycoproteins within the SDFT and CDET, sections were dewaxed, hydrated and stained with Alcian Blue (pH 2.5)/periodic acid Schiff (AB/PAS) using standard staining procedures. Sections were incubated in a 1% Alcian Blue solution (in 3% acetic acid, pH 2.5) for 30 min, washed for 2 min, then treated with 0.5% periodic acid for 10 min. Following a 5‐min wash, sections were incubated with Schiff's reagent for 15 min, washed in running tap water for 5 min and nuclei‐counterstained with Mayer's haemalum for 2 min, before blueing in tap water for 5 min. To determine the distribution of elastic fibres, sections were stained with Miller's elastic stain, and collagen was counterstained with Van Gieson's using standard staining procedures. Sections were treated with 1% potassium permanganate for 5 min, rinsed and then decolorised in 1% oxalic acid for 1 min. Sections were washed in running tap water, rinsed in 95% ethanol and then incubated in Miller's stain for 1.5 h. Sections were rinsed in 95% ethanol, washed in running water, and counterstained with Van Gieson's stain for 4 min. Following staining, sections were dehydrated, cleared and mounted using D.P.X. mountant.

### Immunohistochemistry

Immunohistochemistry was used to assess the distribution of specific proteoglycans within the SDFT and CDET. Sections were de‐waxed, rehydrated and treated with 3% H_2_O_2_ for 10 min to inhibit endogenous peroxidise activity. After pre‐treatment with chondroitinase ABC for 30 min at room temperature [0.1 U mL^−1^ Tris‐buffered saline (TBS)] and blocking [20% goat serum in TBS with 0.05% Tween‐20 (TBS‐T)], samples were washed and primary antibodies were applied at a concentration of 1 : 50 in TBS‐T (see Table 1 for antibody details). Antibodies for decorin, biglycan, lumican and lubricin were a kind gift from Prof. Caterson, Cardiff University, and the fibromodulin antibody was kindly provided by Prof. Roughley, McGill University. Sections were incubated with primary antibody overnight at 4 °C. Negative controls were included where the primary antibody was omitted from the staining procedure. For the mouse monoclonal antibodies, mouse IgG1 (M5284) and IgM (M5909) isotype controls (Sigma‐Aldrich) were included. Following washing, sections were incubated with peroxidase‐conjugated secondary antibodies [goat anti‐mouse IgG (A4416)/IgM (A8786) or goat anti‐rabbit IgG (A6154, all from Sigma‐Aldrich)], with 1 : 50 dilution in 20% goat serum in TBS‐T for 2 h at room temperature. Staining was developed with 3,3′‐diaminobenzidine and nuclei were counterstained with Mayer's haemalum, with the exception of lubricin, where no nuclei‐counterstaining was performed, due to previous studies reporting intracellular staining for lubricin in the Achilles tendon (Sun et al. 2015). Sections were dehydrated and coverslipped with a xylene‐based mountant (DPX). No staining was observed in any negative control samples, confirming that no background staining occurred due to non‐specific binding of the primary or secondary antibodies (Supporting Information Fig. S1).

**Table 1:** Details of antibodies used for immunohistochemical staining

Antibody	Species	Mono/Polyclonal	Epitope recognised	Antibody concentration	Reference
Biglycan (PR8A4)	Mouse IgG	Monoclonal	Core protein	1 : 50	Rees et al. (2000)
Decorin (70.6)	Mouse IgG	Monoclonal	Core protein	1 : 50	Rees et al. (2000)
Fibromodulin (184)	Rabbit	Polyclonal	C‐terminus (CGG)LRLASLIEI	1 : 50	Roughley et al. (1996)
Lubricin (6‐A‐1)	Mouse IgG	Monoclonal	C‐terminal domain	1 : 50	Schumacher et al. (1999)
Lumican	Mouse IgM	Monoclonal	Unknown	1 : 50	

### Image acquisition

Slides were imaged using a digital slide scanner (Nanozoomer, Hamamatsu) and a ×40 objective. Images of each section were acquired using specialist software (ndp.view2) at magnifications ranging from ×5 to ×40. For each stain, one low‐magnification and two high‐magnification images were acquired from transverse and longitudinal sections, resulting in a total of six images from each tendon for each staining condition.

### Image scoring

To assess the distribution of specific proteins, two independent assessors (K.J.K. and J.N.), blinded to tendon type and staining condition, graded the staining intensity of the immunohistochemical images. In each image, the IFM and FM were graded separately on a scale from 0 to 5, with 0 representing no staining and five representing very intense staining. Any images with patchy staining or cutting artefacts were not included in the final analysis. Inter‐observer variability was assessed by calculating linear weighted Kappa statistics (Viera & Garrett, 2005) using an online software tool (http://vassarstats.net/kappa.html).

### Elastic fibre quantification

Elastic fibres were sparsely distributed throughout the IFM and FM, so elastin content was assessed by measuring the length of each fibre and expressing total fibre length relative to IFM or FM area (imagej Software, NIH, USA). Elastic fibre thickness appeared constant across all images and so was approximated to one pixel for all fibres.

### Statistical analysis

To assess differences in staining intensity between paired IFM and FM samples in the SDFT and CDET, repeated measures analysis of variance (anova) were used (prism version 5, GraphPad Software, Inc., La Jolla, CA, USA). To assess differences in staining intensity between tendon types, one‐way anova s were used. Statistical significance was taken as *P* < 0.05.

## Results

### Assessor variation

An overall Kappa statistic of 0.5 was calculated, indicating good agreement between the two blinded observers. There was no difference between Kappa statistics for individual stains.

### General glycoprotein distribution

**Figure 2:**
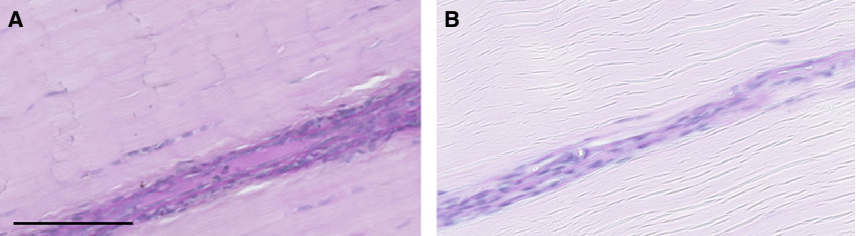
Representative images showing AB/PAS staining for proteoglycans in the SDFT (A) and CDET (B). Scale bar: 100 μm.

Staining of tendon sections using AB/PAS provides information on glycoprotein and proteoglycan distribution, as well as revealing differences in structure between tendon types. Typical AB/PAS images are shown in Fig. 2. It can be seen that tendon structure differs notably between the SDFT and CDET, with a much more distinct and abundant IFM apparent in the SDFT. When considering proteoglycan distribution, there is more intense staining overall in the SDFT than in the CDET. Further, staining is concentrated within the IFM, particularly in the SDFT.

### Decorin distribution

**Figure 3:**
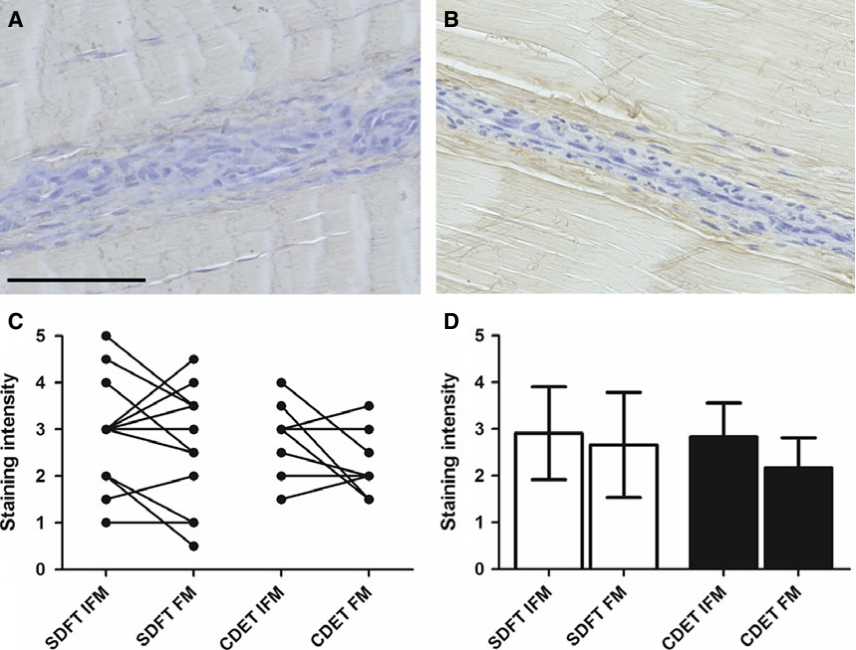
Representative images showing immunohistochemical staining of decorin in the SDFT (A) and CDET (B). Scale bar: 100 μm. There were no significant differences in decorin staining intensity between tendon types or regions (C,D). Individual data points are shown, with lines representing IFM and FM regions in the same image (C). In (D), data are displayed as mean ± SD.

Typical images of decorin staining and resultant scores are shown in Fig. 3. There were no significant differences in decorin staining between tendon type or region.

### Lumican distribution

**Figure 4:**
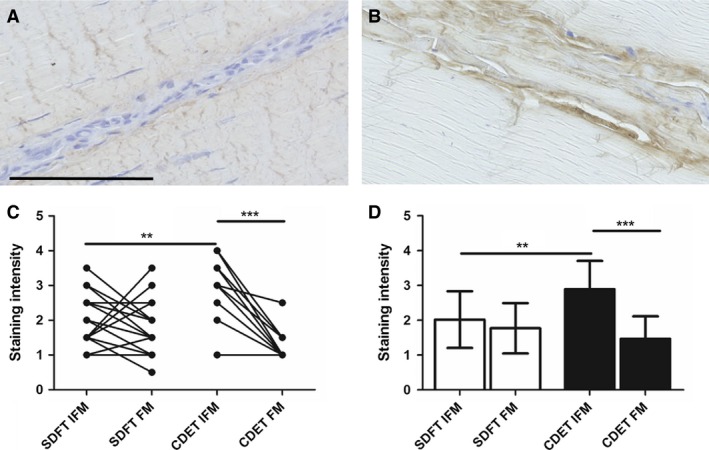
Representative images showing immunohistochemical staining of lumican in the SDFT (A) and CDET (B). Scale bar: 100 μm. Staining intensity was significantly greater in the CDET IFM than in the SDFT IFM and in the CDET FM (C,D). Individual data points are shown, with lines representing IFM and FM regions in the same image (C). In D, data are displayed as mean ± SD. **P < 0.01; ***P < 0.001.

Typical images of lumican distribution and resultant scores are shown in Fig. 4. There was significantly greater staining for lumican in the CDET IFM than in the SDFT IFM (*P* < 0.01). Further, the IFM stained significantly more intensely for lumican than the FM within the CDET (*P* < 0.001).

### Biglycan distribution

**Figure 5:**
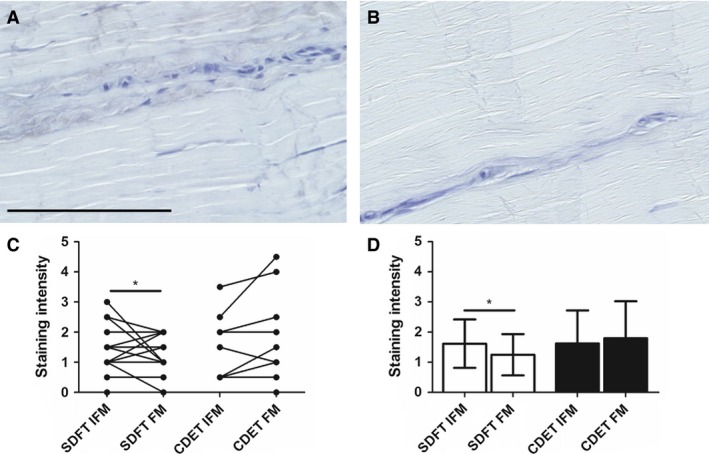
Representative images showing immunohistochemical staining of biglycan in the SDFT (A) and CDET (B). Scale bar: 100 μm. Staining intensity was significantly greater in the SDFT IFM than in the FM (C,D). Individual data points are shown, with lines representing IFM and FM regions in the same image (C). In D, data are displayed as mean ± SD. *P < 0.05.

Typical images of biglycan distribution and resultant scores for staining intensity are shown in Fig. 5. Staining for biglycan was low, and absent in some sections. Where biglycan staining was present, it was often localised to the IFM, with significantly greater staining for biglycan in the SDFT IFM than in the FM (*P* < 0.05).

### Fibromodulin distribution

**Figure 6:**
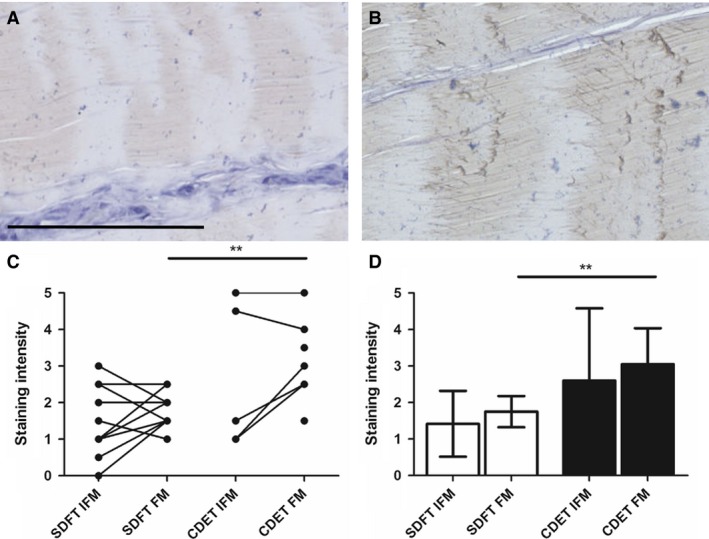
Representative images showing immunohistochemical staining of fibromodulin in the SDFT (A) and CDET (B). Scale bar: 100 μm. Staining intensity was significantly greater in the CDET than in the SDFT FM (C,D). Individual data points are shown, with lines representing IFM and FM regions in the same image (C). In D, data are displayed as mean ± SD. **P < 0.01.

Typical images showing fibromodulin distribution and scores for staining intensity are shown in Fig. 6. Fibromodulin staining appeared more intense in the CDET than in the SDFT, and there was significantly greater staining in the CDET FM than in the SDFT FM (*P* < 0.01).

### Lubricin distribution

**Figure 7:**
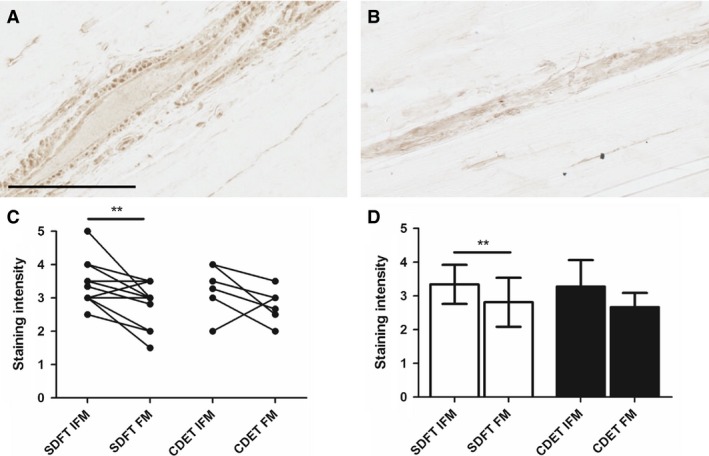
Representative images showing immunohistochemical staining of lubricin in the SDFT (A) and CDET (B). Scale bar: 100 μm. Staining intensity was significantly greater in the SDFT IFM than in the FM (C,D). Individual data points are shown, with lines representing IFM and FM regions in the same image (C). In D, data are displayed as mean ± SD. **P < 0.01.

Typical images showing lubricin distribution are shown in Fig. 7. Lubricin was predominantly localised to the IFM in both the SDFT and the CDET, with significantly greater staining in the SDFT IFM than in the FM (*P* < 0.01).

### Elastin distribution

**Figure 8:**
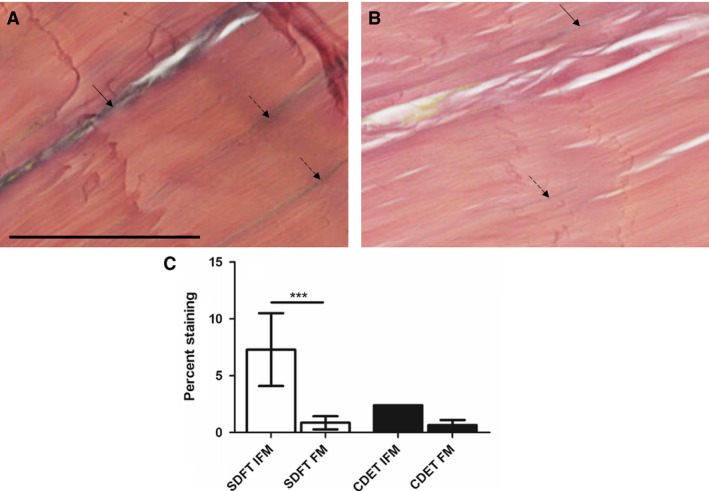
Representative images showing Elastic von Gieson's staining of the SDFT (A) and CDET (B). Scale bar: 50 μm. Elastic fibres are visible as blue/black lines. In the SDFT, elastin staining was predominantly localised to the IFM (arrow) with a small number of elastic fibres evident within the FM (dashed arrows). In the CDET, a small number of elastic fibres were identified in the IFM and in the FM. Quantification of the percent staining of elastin demonstrated significantly greater elastin in the SDFT IFM than in the FM (C). It was not possible to perform statistical analyses comparing the SDFT and CDET due to low numbers of stained elastic fibres in the CDET groups. ***P < 0.001.

Typical images showing the distribution of the elastic fibres and percentage staining are shown in Fig. 8. It was not possible to assess significant differences in percentage staining between the SDFT and CDET due to the low numbers of CDET samples in which elastin was present. In the SDFT, elastic fibres were predominantly located within the IFM, with significantly greater percentage staining in the IFM than in the FM (*P* < 0.001).

## Discussion

In this study, we have assessed the distribution of extracellular matrix proteins in different tendon compartments and between tendon types. The data support our hypothesis, showing a different distribution of proteoglycans and elastin in the functionally distinct SDFT and CDET. Further, we have shown that the distribution of specific proteins varies with tendon region, with localisation of lubricin and elastin to the IFM, particularly in the energy‐storing SDFT.

The finding that elastic fibres are localised to the IFM in the SDFT, supports previous studies that have demonstrated that the IFM is rich in elastin, both in tendon (Grant et al. 2013) and ligament (Smith et al. 2011). However, elastin was largely absent in the CDET IFM. Our previous work has demonstrated that the IFM in the SDFT is more extensible and elastic than the CDET IFM (Thorpe et al. 2012, 2015b). Taken together, these data suggest that elastin may play an important role in tendon recoil, enabling the greater interfascicular recoil that is seen in energy‐storing tendons (Thorpe et al. 2015b).

General proteoglycan and glycoprotein staining was greater in the SDFT than in the CDET, which supports previous studies showing a greater glycosaminoglycan content in the SDFT (Batson et al. 2003; Thorpe et al. 2010). Further, overall proteoglycan staining was more intense in the IFM than in the FM in both tendon types. When considering the distribution of specific proteoglycans, lubricin was found to be localised to the IFM in both tendon types. This supports previous studies that have shown localisation of lubricin to the IFM in the human Achilles (Sun et al. 2015), goat supraspinatus (Funakoshi et al. 2008) and mouse tail (Kohrs et al. 2011) tendons, as well as to the tendon sheath (Taguchi et al. 2009). Further, it has been demonstrated that a reduction in lubricin content reduces tendon and fascicle gliding ability (Taguchi et al. 2009; Kohrs et al. 2011), suggesting that the function of this glycoprotein in the IFM may be to facilitate sliding between fascicles. This is particularly important in energy‐storing tendons, where the greater requirement for extension appears to be provided by a larger degree of interfascicular sliding (Thorpe et al. 2012). The presence of intracellular lubricin staining was also observed in interfascicular and fascicular cells in both tendon types. The presence of intracellular lubricin has been reported previously in tendon (Sun et al. 2015), intervertebral disc (Shine & Spector, 2008) and the superficial layer of cartilage (Schumacher et al. 1999). It is not clear whether this staining represents production of lubricin that will be secreted into the matrix, or whether lubricin has an additional intracellular function. To date, several isoforms of lubricin have been identified, and lubricin can occur in both proteoglycan and glycoprotein forms (Lord et al. 2011). It has been suggested that these different isoforms may have distinct functions, including lubrication, matrix‐binding, cytoprotection and cell proliferation (Flannery et al. 1999).

The differential distribution of SLRPs identified between tendon types and regions may indicate a distinct role for each of these proteoglycans in tendon. Decorin is the most abundant proteoglycan within tendon (Yoon & Halper, 2005) and plays an important role in collagen fibrillogenesis during development (Birk et al. 1995). Decorin distribution was not significantly different between the IFM and FM in either tendon type, supporting a previous study that showed decorin staining throughout the IFM and FM in the SDFT (Kim et al. 2010). While biglycan is present at a lower abundance than decorin in tendon, it is also a regulator of fibrillogenesis (Zhang et al. 2006). Fibromodulin and lumican are also involved in collagen fibrillogenesis (Ezura et al. 2000) and fibromodulin has an additional role in modulating collagen crosslink formation (Kalamajski et al. 2014). The role of the SLRPs in adult tendon is yet to be fully elucidated, although data from several studies suggest they may contribute to tendon mechanical properties. Studies in knock‐out mice have reported that decorin and biglycan modulate tendon viscoelasticity (Robinson et al. 2005; Dourte et al. 2012), and that this response is tendon‐specific (Robinson et al. 2005). Further, a recent study has demonstrated that enzymatic disruption of chondroitin sulphate side chains reduces sliding between collagen fibrils (Rigozzi et al. 2013), suggesting that decorin and biglycan, which have chondroitin sulphate side chains, may facilitate inter‐fibril sliding and contribute to fascicle extension. In the current study we have shown that these SLRPs are also present within the IFM, which indicates they may also contribute to sliding between fascicles.

Additionally, SLRPs may play a non‐mechanical role in the IFM. While it has previously been thought that the IFM is composed predominantly of non‐collagenous proteins, recent profiling of the IFM proteome identified the presence of 10 types of collagen in the IFM, (Thorpe et al. 2016), which appear to be turned over more rapidly than collagen within the FM (Thorpe et al. 2015a). The presence of SLRPs within the IFM may therefore be required for regulation of ongoing collagen fibrillogenesis in this compartment. Further studies are required to establish how specific SLRPs contribute to tendon function and how this may vary between tendon types.

In contrast to the results in the current study, we have previously, using mass spectrometry and Western blotting techniques, reported that both decorin and fibromodulin are more abundant in the FM than in the IFM in the SDFT (Thorpe et al. 2016). However, mass spectrometry and Western blotting are more sensitive than immunohistochemical techniques and therefore may identify differences that are not apparent in the current study. Further, our previous analysis of the IFM and FM proteome did not identify elastin or lubricin in either compartment. However, this is likely due to inherent difficulties in identifying these proteins using mass spectrometry (Thorpe et al. 2016). The specificity of the antibodies used in the current study must also be considered. With the exception of fibromodulin, all antibodies used were mouse monoclonal antibodies, and no staining was observed when using appropriate isotype controls, providing confidence that there was no non‐specific background staining. Further, previous studies have validated the specificity of the decorin and biglycan antibodies by pre‐adsorption with purified protein (Rees et al. 2000). Although it is not possible to perform isotype control experiments for the fibromodulin antibody as this is a rabbit polyclonal antibody, the specificity of this antibody has been determined previously by pretreating an antibody preparation with the immunising peptide, which abolished the staining (Roughley et al. 1996). It should be noted that these pre‐adsorption studies were performed in species different to that used in the current study; however, it is unlikely that antibody specificity will be markedly affected by species differences and we are therefore confident in the specificity of the antibodies used. Using immunohistochemical methods, it is not possible to compare abundance of different proteins, and protein amounts can only be assessed semi‐quantitatively. It should also be noted that staining intensity is not directly proportional to protein abundance and comparison between proteoglycan types is not possible. Future studies should therefore establish the absolute amounts of individual proteins within the IFM and FM of functionally distinct tendons.

## Conclusions

We have demonstrated for the first time how the distribution of specific proteins differs between tendon type and sub‐structural compartments. Lubricin is enriched in the IFM, which likely facilitates interfascicular sliding, a function that is particularly important in energy‐storing tendons. We have also shown that elastin is localised to the IFM in energy‐storing tendons, which may enable the greater elasticity and ability to recoil seen in this tendon type. Further, we identified differential distributions of SLRPs within tendon. These data provide important advances into fully characterising structure–function relationships within tendon.

## Author contributions

CTT, GPR, HLB, PDC and HRCS designed the study, CTT acquired and interpreted data, CTT, KK and JN analysed data, CTT drafted the manuscript, GPR, HLB, PDC and HRCS revised the manuscript.

## Supporting information


**Fig. S1.** Images of negative isotype controls, in which the primary antibody has been replaced by a mouse IgG1 (a) and IgM (b) isotype control antibodies. Sections from the SDFT were counterstained with Mayer's haemalum. Scale bar: 100 μm.
