# Supplementary figures and images for: Distribution of proteins within different compartments of tendon varies according to tendon type
Source: J Anat. 2016 Apr 25;229(3):450–8. doi: 10.1111/joa.12485 (PMC4974547; doi:10.1111/joa.12485)

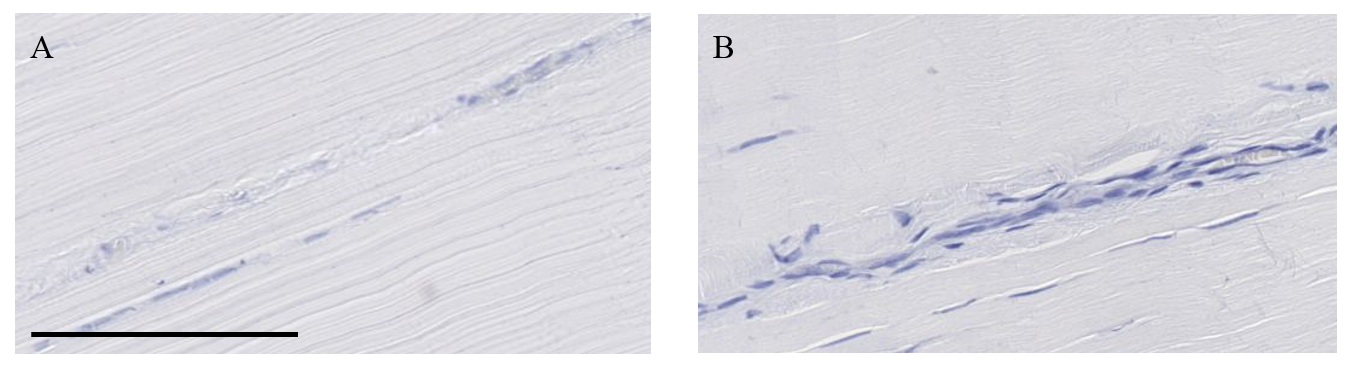

Supplement: Supplementary file 1 — Fig. S1. Images of negative isotype controls, in which the primary antibody has been replaced by a mouse IgG1 (a) and IgM (b) isotype control antibodies. Sections from the SDFT were counterstained with Mayer's haemalum. Scale bar: 100 μm. [file JOA-229-450-s001.tif]
